# Effect of table inclination angle on videolaryngoscopy and direct laryngoscopy: Operator’s muscle activation and laryngeal exposure analysis

**DOI:** 10.1186/s12871-022-01849-5

**Published:** 2022-10-03

**Authors:** Efrain Riveros-Perez, Lori Bolgla, Nianlan Yang, Bibiana Avella-Molano, Camila Albo, Alexander Rocuts

**Affiliations:** 1 Department of Anesthesiology and Perioperative Medicine, Medical College of Georgia at Augusta University, 1120 15th street BI-2144, Augusta, GA 30912 USA; 2 College of Allied Health Sciences, Augusta University, Augusta, GA USA; 3 Department of Anesthesiology and Perioperative Medicine, Medical College of Georgia at Augusta University, GA Augusta, USA; 4 Medical College of Georgia at Augusta University, Augusta, GA USA

**Keywords:** Intratracheal intubation, Airway management, Electromyography

## Abstract

**Background:**

Optimal vocal cord visualization depends on the patient’s anatomical factors, characteristics of the laryngoscope, and the operator’s muscle action. This study evaluated the effect of table inclination and three different laryngoscopic methods on procedural variables. The primary aim of this study is to compare differences in laryngoscopic view among clinicians based on the instrument used and table orientation. The secondary aim is to determine differences in upper extremity muscle activity based on laryngoscope use and table inclination.

**Methods:**

Fifty-five anesthesia providers with different experience levels performed intubations on a manikin using three angles of table inclination and three laryngoscopy methods. Time to intubation, use of optimization maneuvers, glottic view, operator’s comfort level, and upper extremity muscle activation measured by surface electromyography were evaluated.

**Results:**

Table inclination of 15° and 30° significantly reduced intubation time and the need for optimization maneuvers. Fifteen degrees inclination gave the highest comfort level. Anterior deltoid muscle intensity was decreased when table inclination at 15° and 30° was compared to a flat position.

**Conclusion:**

Table inclination of 15° reduces intubation time and the need to use optimization maneuvers and is associated with higher operator’s comfort levels than 0° and 30° inclination in a simulated scenario using a manikin. Different upper extremity muscle groups are activated during laryngoscopy, with the anterior deltoid muscle exhibiting significantly higher activation levels with direct laryngoscopy at zero-degree table inclination.

## Introduction

The primary purpose of conventional laryngoscope blades is to align the patient’s airway axes with the operator’s line of sight. Traditionally, clinicians have used the Macintosh blade, which may be challenging for novice personnel [1]. Insufficient axes alignment can make endotracheal tube advancement cumbersome. Videolaryngoscope technology has been developed to improve glottis visualization without the need to align all axes [2, 3]. Complications like damage to the soft palate, palatopharyngeal and palatoglossal arches have been associated with videolaryngoscope use [4].

The operating table is an important yet sometimes overlooked piece of equipment necessary for tracheal intubation. The height of the table in relation to the operator’s position affects ergonomics and laryngoscopic view [5–7]. Upper limb muscle activity generated during intubation is another variable that deserves consideration. Increased muscle activity, as quantified using surface electromyography (EMG), is associated with decreased comfort and performance [4–6].

The purpose of this investigation was two-fold. The first purpose was to compare differences in laryngoscopic view among clinicians who currently perform or may perform laryngoscopy based on instrument use and table orientation. The second purpose was to determine differences in upper extremity muscle activity based on the laryngoscopic type used (direct laryngoscopy, indirect laryngoscopy with hyper angulated blade and Macintosh-like blade) and table inclination.

## Methods

This is a pilot study. After ethical approval by the Augusta University Institutional Review Board, all the methods were performed in accordance with relevant guidelines and regulations. This study has been reported according to the STROBE criteria [8]. Fourth-year medical students, anesthesiology residents, attending anesthesiologists, and certified nurse anesthetists were invited to participate in the study. Those with musculoskeletal injuries or limitations affecting the left upper extremity were excluded. Participants signified their voluntary intent to participate by signing a university-approved informed consent document. No additional education in laryngoscopy was provided to participants for the sole purpose of this study.

### Laryngoscopy procedures

Each participant performed direct laryngoscopy (DL) with a Macintosh blade No.3, indirect laryngoscopy with a hyper angulated blade (Glidescope®,Verathon Medical, Bothell, WA, USA), and a Macintosh-like blade (C-MAC®,Karl Storz, Tuttlingen, Germany) to intubate a manikin (SimMan® Essential, ALS simulator, Laerdal) with an endotracheal tube size 7.0 with stylet. The manikin allowed adjustment of head position. The laryngoscopy procedures were performed in the flat position (0°) and in back-up positions at 15^o^ and 30°. The participant was asked to obtain the best possible laryngoscopic view before intubation. The position of the table was determined with an inclinometer attached to the trunk part of the bed. The operator was in the standing position.

The primary outcomes were laryngoscopic view grade measured by the percentage of glottis opening (POGO) scale [9]. The POGO was defined by the linear span from the anterior commissure to the inter-arytenoid notch [10]. Subjects provided a self-perceived POGO score (POGO-subj) for each intubation which was then scored by one of the subinvestigators (POGO-obj). Measured secondary outcomes were time to intubation from start of laryngoscopy until the tracheal tube passed the vocal cords, number of intubation attempts, use of optimization maneuvers, and subjective level of discomfort measured with a visual analogue scale (1 = full comfort, 10 = full discomfort).

### EMG Procedures

The operator’s left upper extremity was instrumented with surface EMG electrodes. An investigator prepared the subject’s skin for the surface EMG electrodes by shaving (if needed) and cleaning the skin with isopropyl alcohol over the following muscles: 1) anterior deltoid; 2) posterior deltoid; 3) biceps brachii; 4) flexor carpi radialis; and 5) brachioradialis. All electrodes were placed over the muscle belly, and activity was confirmed by visualization via an oscilloscope.

Subjects performed three maximum voluntary isometric contractions (MVIC) for each muscle to enable normalization of the EMG data [11]. For this purpose, subjects generated a maximum contraction over 2 s and held this contraction for an additional 5-s period. Subjects rested for 30 s between each MVIC. To determine the MVIC for each muscle, we positioned subjects in a manner consistent with manual muscle techniques used by physical therapists. Subjects also performed one sub-maximal (50% effort) practice trial prior to data collection. For data collection, each subject was instructed to begin after the investigator’s signal. Subsequently, a trigger switch was activated and held until intubation completion. The EMG activity collected during this time period was processed for analysis.

### EMG instrumentation and analysis

An 8-channel wireless EMG system (Delsys™, Boston, MA) collected all EMG data. EMG data were sampled at 2000 Hz and band-passed filtered between 20 and 450 Hz. Unit specifications include a common mode rejection ratio greater than 80 dB. All data was root-mean-squared (RMS) over a 30-ms moving window. For the MVICs, a computer algorithm determined the maximum RMS amplitude across a moving 500-ms average window across each MVIC. The highest amplitude window represented 100% MVIC (% MVIC). The average amplitude of EMG data during each laryngoscopy view was calculated, expressed as a % MVIC, and used for statistical analysis.

### Statistical analysis

All statistical analyses were performed using SAS 9.4. Statistical significance was assessed using an alpha level of 0.05. After evaluating normal distribution of the data with normal quantile plot and Shapiro–Wilk test, appropriate descriptive statistics (group means and standard deviations) were calculated for POGO scores (subj and obj) and EMG data. Repeated measures analysis of variance (ANOVA) was used to determine whether different angles or laryngoscopy methods affected POGO scores and EMG data. Analysis of activity across blade, table position, and experience level was done using the Bonferroni-Holm correction. Originally, we ran a 3 (blades) X 3 (table position) X 3 (experience) mixed-model ANOVA with repeated measures. Bonferroni-adjusted comparisons were performed to examine post hoc pairwise comparisons.

## Results

The study population consisted of fifty-five subjects: medical students (*n* = 12), postgraduate year-one (PGY-1) anesthesia residents (*n* = 2), CA-1 residents (*n* = 10), CA-2 residents (*n* = 6), CA-3 residents (*n* = 11), anesthesiologists (*n* = 9) and nurse anesthetists (*n* = 5). All participants (thirty male and twenty-five female) performed procedures with direct laryngoscope, C-MAC® videolaryngoscope and Glidescope®, at the three levels of table inclination.

### Effect of table inclination

**Fig.1:**
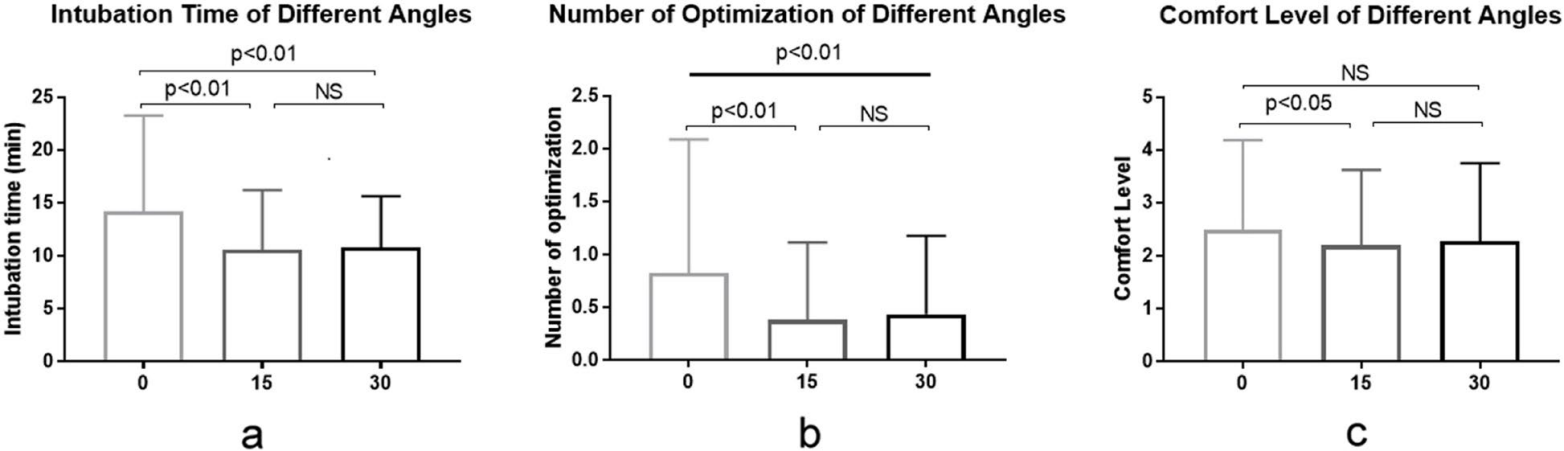
Means and standard deviations for the effect of table inclination angles on (**a**) intubation time, (**b**) use of optimization maneuvers, (**c**) and operator’s comfort level

Compared to a zero-degree table position, 15 and 30 degrees of inclination significantly reduced intubation time (*p* < 0.01) and the need to use optimization maneuvers (*p* < 0.01). An inclination of 15 degrees provided the highest comfort level compared to that obtained at 0 and 30 degrees, with a statistically significant difference between 15 and 0 degrees(Fig. 1). Anterior deltoid muscle activation intensity varied inversely with table inclination from zero-degree to 15 and 30 degrees, respectively; with a statistically significant difference between zero and 30 degrees (*p*-value 0.02) (Table 1).

**Table 1:** Effect of table inclination on laryngoscopic variables

Table inclination (degrees)	Intubation time (seconds)		Optimization maneuvers (number)		MVIC (%)		Comfort level (number)	
	Mean	SD	Mean	SD	Mean	SD	Mean	SD
0	14.18	9.09	0.83	1.26	27.50	20.53	2.5	1.73
15	10.62	5.60	0.38	0.74	24.54	15.35	2.18	1.47
30	10.83	4.80	0.44	0.74	23.28	13.58	2.26	1.52

Overall values for all levels of experience

*MVIC* Maximum voluntary isometric contraction, *SD* Standard deviation

### Effect of method of laryngoscopy

**Fig. 2:**
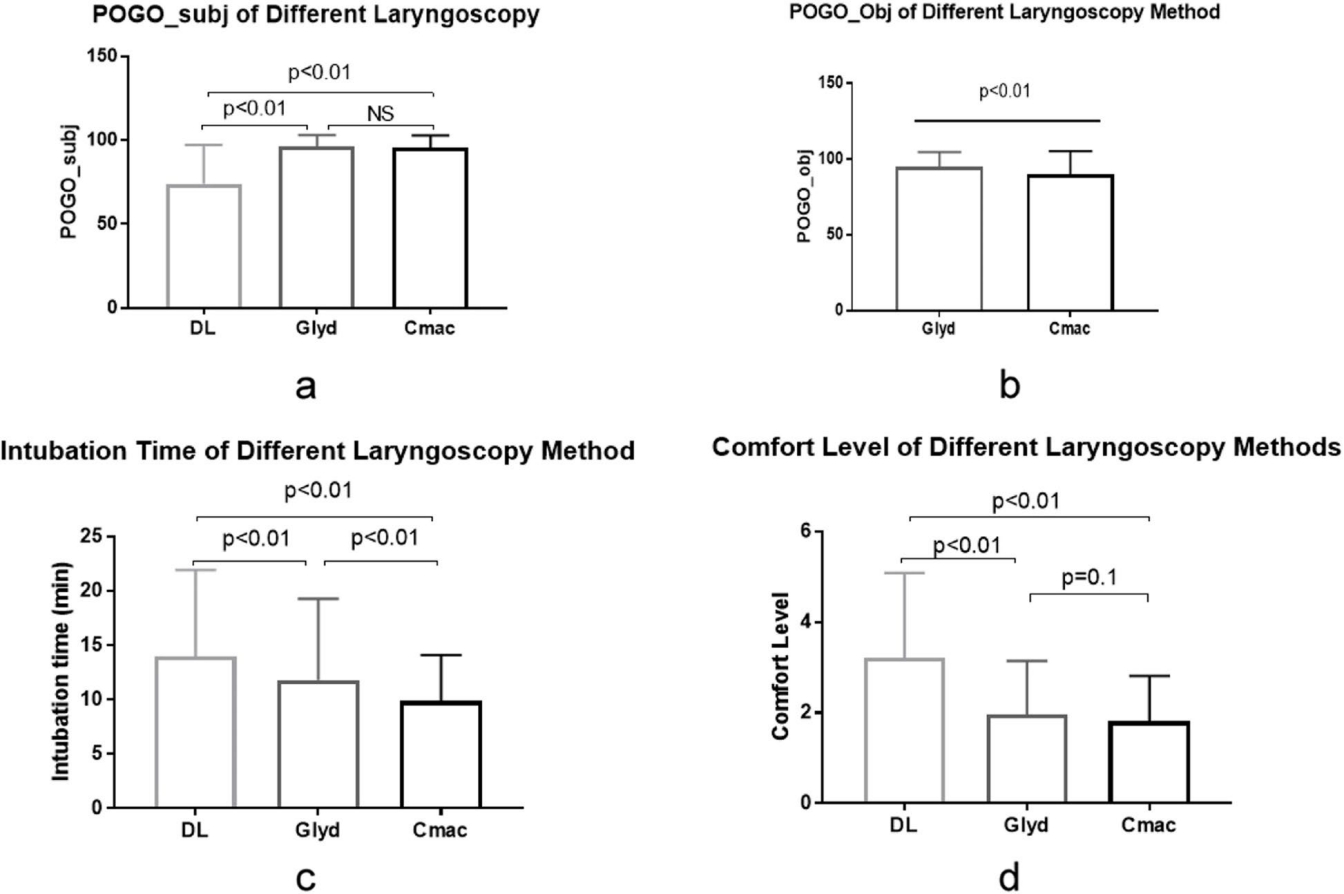
Means and standard deviations for the effect of different laryngoscopy methods on vocal cord view. (**a**) subjective POGO, (**b**) objective POGO, (**c**) intubation time, and (**d**) operator’s comfort level. POGO, percentage of glottis opening. DL, direct laryngoscope. Glyd, Glidescope. Subj, subjective. Obj, objective. NS, non-significant

Compared with C-MAC® and Glidescope®, DL exhibited the lowest POGO-subj score (*p* < 0.01), the lowest comfort level (*p* < 0.01), the longest intubation time (*p* < 0.01) and overall highest muscle activation intensity. Subjects also demonstrated greater POGO-obj scores and intubation times with the Glidescope® (*p* < 0.01) than C-MAC® (Fig. 2).

### Combined effect of table inclination and laryngoscopy method

**Fig. 3:**
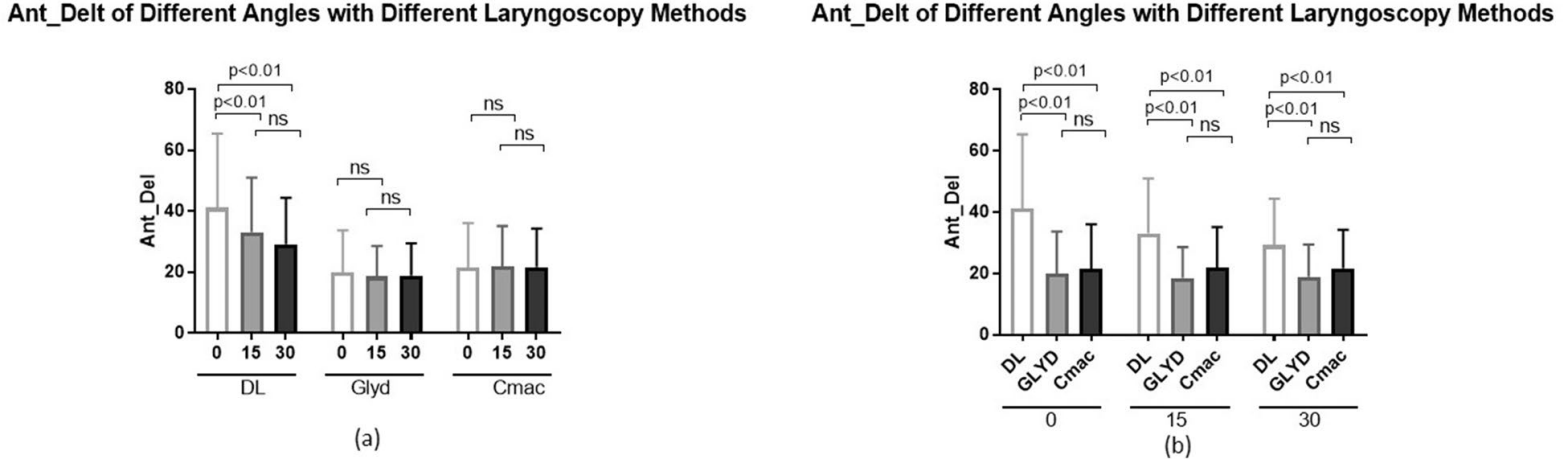
Means and standard deviations for the effect of table inclination (**a**) and laryngoscopy methods (**b**) on anterior deltoid muscle activity. Ant_Delt, anterior deltoid. DL, direct laryngoscopy. Glyd, Glidescope. NS, non-significant

The degree of table inclination and laryngoscopy method affected muscle activity intensity of the anterior deltoid muscle. The two factors exhibited meaningful interaction; that is, the effect of table inclination on anterior deltoid muscle activity intensity was different when different laryngoscopes were used. Comparing the mean anterior deltoid muscle activity using DL, a statistically significant difference was evidenced between zero and 15 and 30 degrees of table inclination. A statistically significant difference between DL and both types of indirect laryngoscopy was shown when the three degrees of inclination were analyzed separately (0, 15, and 30 degrees) (Fig. 3).

**Fig. 4:**
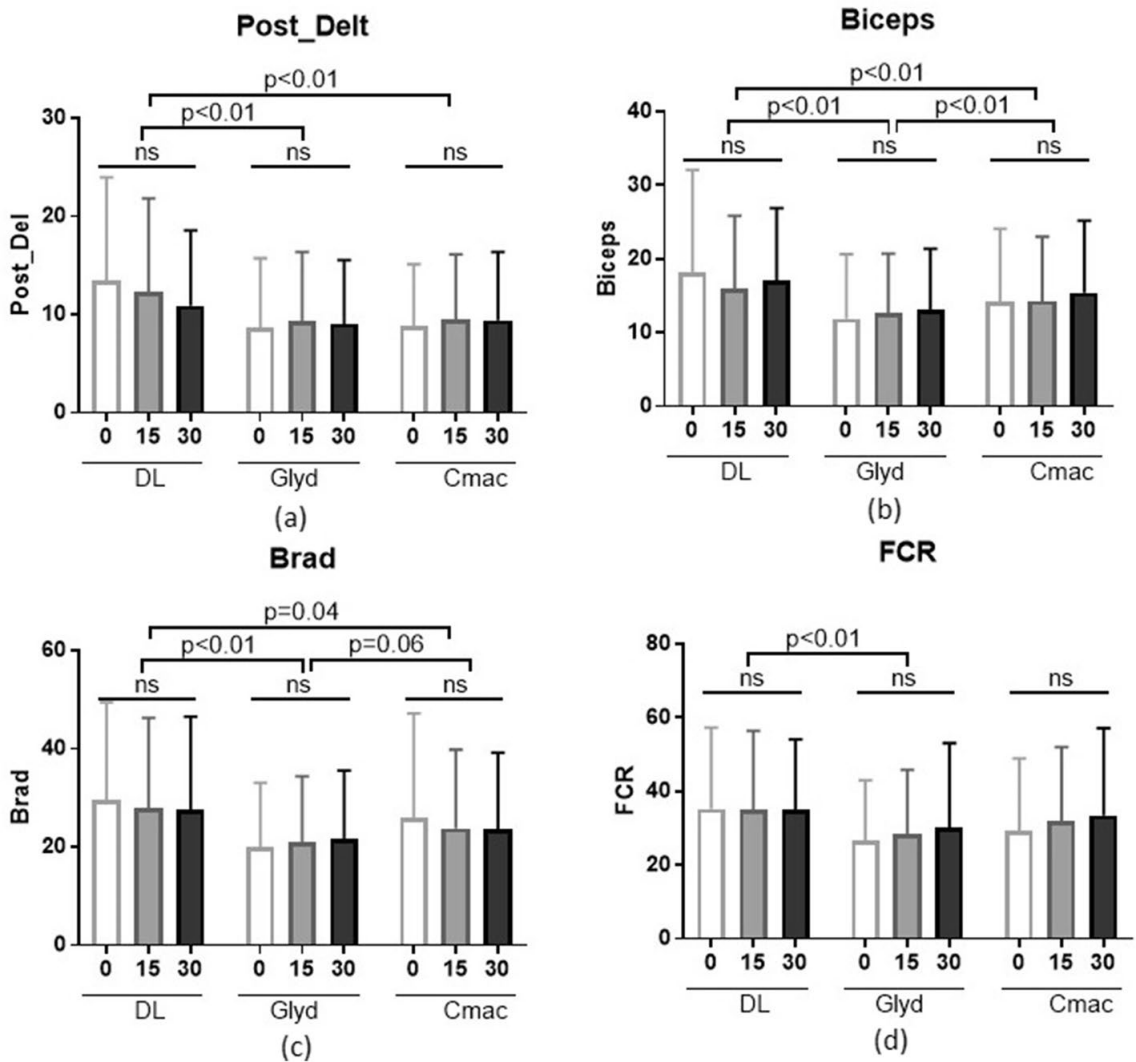
Means and standard deviations for the effect of different laryngoscopy methods on muscle activity. Posterior deltoid (**a)**. Biceps brachii (**b**). Brachioradialis (**c**). Flexor carpi radialis (**d**). DL, direct laryngoscopy. Glyd, Glidescope. Post_Delt, posterior deltoid. Brad, brachioradialis. FCR, flexor carpi radialis. NS, non-significant

On the other hand, table inclination showed no significant effect on activity intensity of posterior deltoid, biceps brachii, brachioradialis, and flexor carpi radialis muscles within each group using the same laryngoscopic method. DL was associated with significantly higher posterior deltoid and biceps muscle activity compared to the other two methods of laryngoscopy; higher brachioradialis and flexor carpi radialis muscle activity compared to Glidescope. Likewise, there was a statistically significant difference in the mean biceps muscle activity between Glidescope and C-MAC (Fig. 4).

**Fig. 5:**
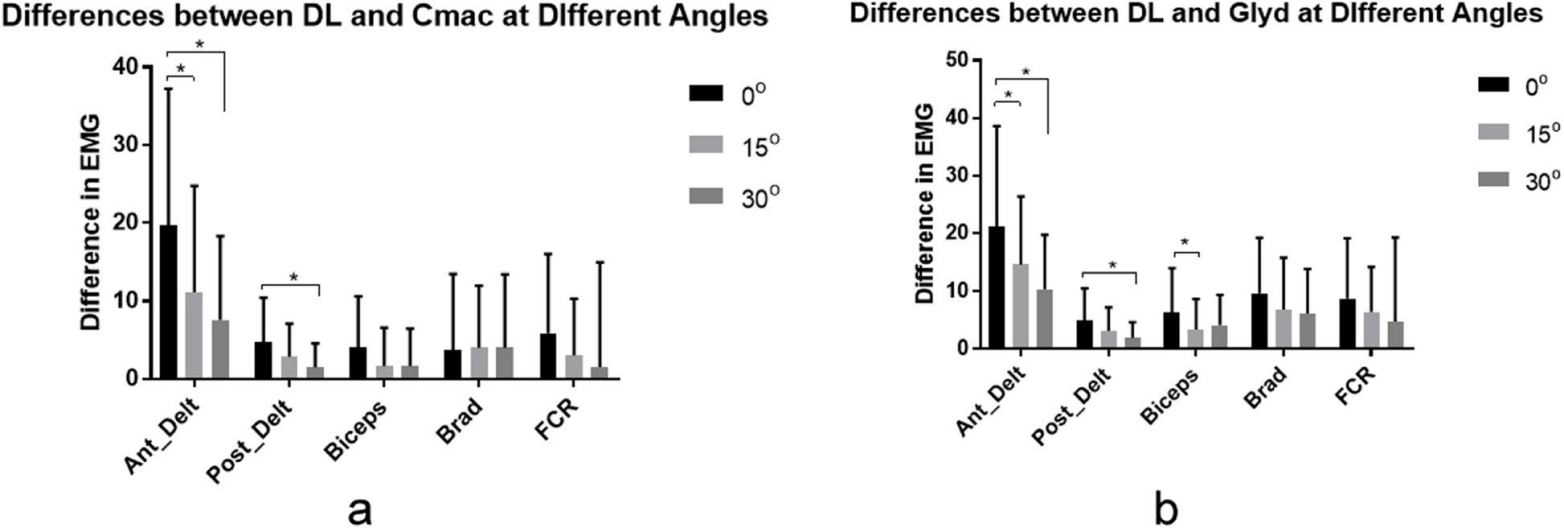
Differences in muscle activity in relation to laryngoscope. DL vs. C-MAC (**a**). DL vs. Glidescope (**b**). Ant_Delt, anterior deltoid. Post_Delt, posterior deltoid. Brad, brachioradialis. FCR, flexor carpi ulnaris. DL, direct laryngoscope. Glyd, Glidescope

The differences in muscle intensity between different laryngoscopy methods were closer to the established significance level when table inclination was closer to zero-degree. A statistically significant difference in muscle intensity was evidenced between zero-degree and 15 and 30 degrees for the anterior deltoid muscle and between zero and 30 degrees for the posterior deltoid muscle when comparing DL and both indirect laryngoscopy techniques. Furthermore, a statistically significant difference in muscle intensity was shown between zero and 15 degrees for the biceps muscle when comparing DL with Glidescope (Fig. 5).

### Effect of years of experience

There were significant differences in intubation time among different groups of participants based on years of experience. Pair-wise comparisons revealed that PGY-1 and CA-1 residents had longer intubation times compared to other groups (Table 2).

**Table 2:** Intubation time per level of training

Training level	Intubation time (seconds)	
	Mean	Standard deviation
Medical student	12.4	5.7
PGY-1	16.3	7.8
CA-1	15.7	8.0
CA-2	10.2	4.8
CA-3	10.6	7.3
CRNA	9.7	4.7
Attending	9.8	6.7

*PGY* Postgraduate year, *CA* Clinical anesthesia year, *CRNA* Certified registered nurse anesthetist

### Correlation analysis

POGO (subj and obj) scores were correlated with level of experience, laryngoscopy method, use of optimization maneuvers (*p* < 0.0001), and number of attempts (0.014) but was not correlated with table inclination. In addition, intubation time showed correlation with experience level, laryngoscopy method, and table inclination (*p* < 0.0001). Regarding the muscle groups, anterior deltoid activity was correlated with participant group (experience level), laryngoscopy method, and table inclination (*p* < 0.0001).

### Average EMG Activity across blade, table position, and experience level

We found interaction effect between blade and table position. The anterior deltoid muscle exhibited greater activation with DL compared with Glidescope® and C-MAC® at all angles (0° = 41.1, 15° = 33.1, and 30° = 31.8% MVIC) (Table 3).

**Table 3:** Anterior deltoid muscle activation comparing 15 and 30 degrees of table inclination between laryngoscopy methods

Table inclination (degrees)	Glidescope MVIC (%)	C-MAC MVIC (%)	DL MVIC (%)
15	18.5	22.2	33.1
30	18.9	22.9	31.8

*DL* Direct laryngoscopy, *MVIC* Maximum voluntary isometric contraction

**Fig. 6:**
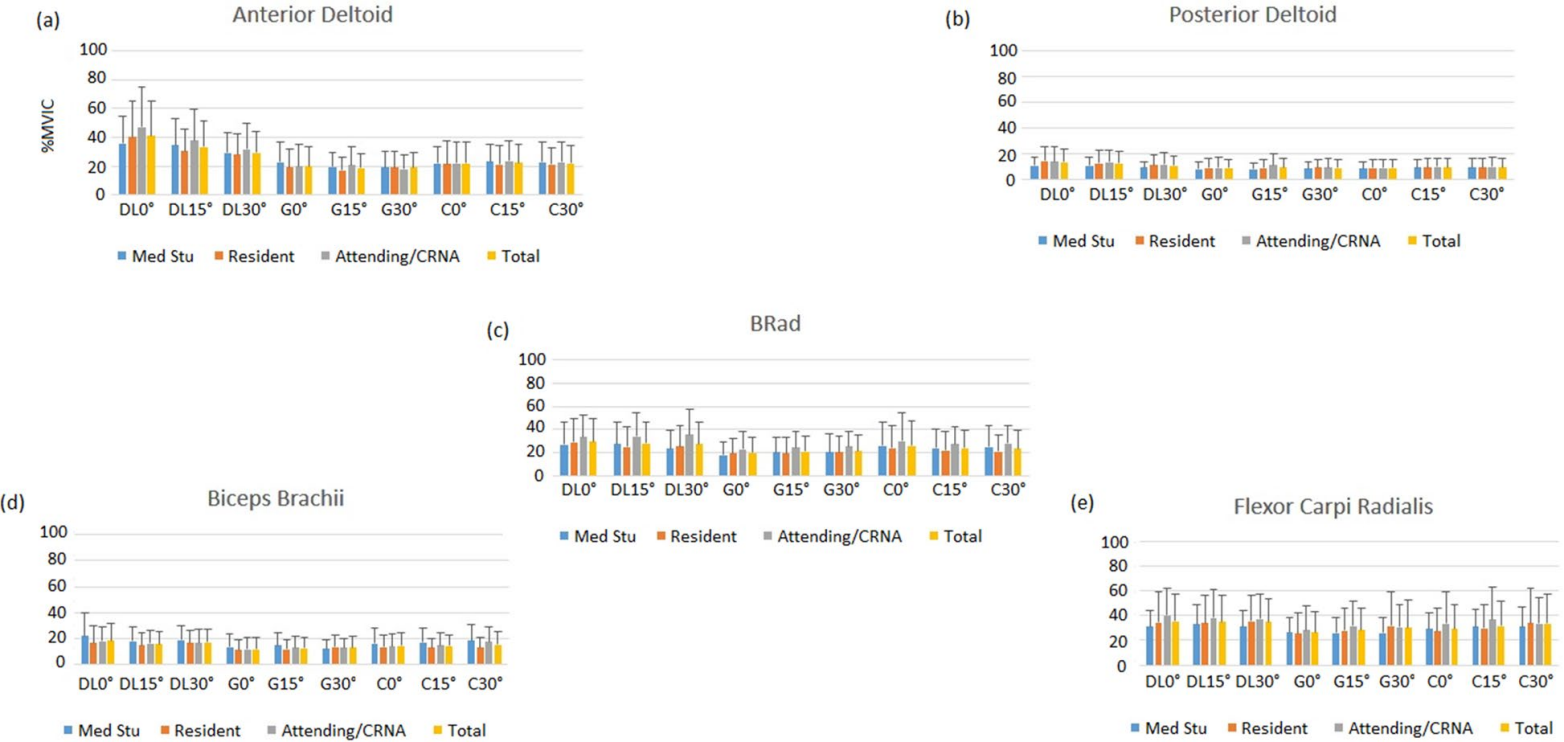
Summary of Average EMG Activity across blade, table position, and experience level. Anterior deltoid (**a**). Posterior deltoid (**b**). Brachiradialis (**c**). Biceps Brachii (**d**). Flexor Carpi Ulnaris (**e**). MVIC%, maximum voluntary isometric contractions. Med Stud, medical student. Attend, attending anesthesiologist. CRNA, nurse anesthetist. DL, direct laryngoscopy. G, GlideScope. C, C-MAC videolaryngoscope

The posterior deltoid muscle activation was greater with DL at all angles. Activation levels for DL ranged from 10.5 to 13.9% MVIC, which are low and not clinically relevant. For the biceps brachii muscle, DL had greater activation (17.1 to 18.3% MVIC) than Glidescope® (11.9 to 13.0% MVIC) at all angles and C-MAC® at zero-degree. C-MAC® had greater activation (14.2 to 15.4% MVIC) than the Glidescope® at all angles. Overall, activation levels were low and not clinically relevant but showed a pattern of less biceps activity required for Glidescope®. Concerning flexor carpi radialis, only a main effect on blade was observed (table position and experience level had no effect). DL was associated with greater activation (35.0 to 35.3% MVIC) than other methods. This muscle showed a similar pattern of less EMG activity generated with the three laryngoscopy methods. Finally, the brachioradialis muscle exhibited a similar pattern to flexor carpi radialis, with DL showing greater activation (27.6 to 29.6% MVIC) than videolaryngoscopy (Fig. 6).

## Discussion

Laryngoscopic procedures involve the interaction between patient’s anatomical features, characteristics of the device used, and operator’s musculoskeletal factors. Our study findings support the notion that using a table inclination of 15 degrees is associated with lower intubation time and a decreased need to use optimization maneuvers; this difference was statistically significant when comparing it against a zero-degree table inclination. In addition, anterior deltoid muscle electrical activity intensity as a surrogate of operator’s upper extremity muscle activation increases when DL is used compared to videolaryngoscopy at different degrees of table inclination. Furthermore, the observed muscle activity that occurs with DL is more accentuated when the table is in a flat position.

An inclination of 15 degrees provided the highest comfort level compared to other levels of inclination. When table inclination level is combined with laryngoscopy method, we observed that muscle activation (anterior deltoid) was greatest at zero-degree table inclination and with direct laryngoscopy. We may argue that the use of Glidescope® with a table inclination of 15 degrees provides favorable conditions for laryngoscopy.

Most studies exploring the dynamics of DL have focused on patient anatomical aspects and the three axes alignment theory [12, 13]. Table inclination is an often-overlooked contributor to the interplay of physical variables during laryngoscopy. Our results are consistent with Turner’s findings of higher first-pass success rates of intubation when some degree of table inclination was employed, as we evidenced that DL at 15 and 30 degrees are associated with lower intubation times and less need to use additional optimization maneuvers while improving the level of comfort of the operator [14].

We also found that the operator’s upper extremity muscle activation (anterior deltoid) was more significant with DL compared with videolaryngoscopic methods. The explanation for this finding might lie in differences in design between the devices. The Glidescope® allows patient's intubation without the need to achieve perfect axis alignment which might lead to less muscle activation by the proceduralist [15]. On the other hand, the C-MAC® videolaryngoscope mounted camera provides a better view that makes muscle activation less necessary to obtain an adequate glottic view.

Regarding POGO,direct laryngoscopy had a lower POGO-subj than the Glidescope® and C-MAC®. Interestingly, the Glidescope® and C-MAC® had similar POGO-subj but different POGO-obj scores. While different from a statistical standpoint, Glidescope® POGO-obj was only 5.6% greater than C-MAC®. This finding likely has minimal clinical significance. In summary, the glottis visualization was related to optimization maneuvers to align airway axes and experience level, but not with table inclination. We may argue that optimal patient positioning and an experienced operator to improve the chance of successful intubation must accompany the 15-degree table inclination.

Skeletal muscle electrical activity and contraction are physiologically linked processes. EMG is commonly used to infer the pattern of muscle contraction; however, factors such as muscle length, muscle velocity, and type of fiber influence the relationship between electrical and mechanical activity [16]. Non-linear force-EMG relationships occur at low levels of muscle activity. In addition, passive structures not evaluated by EMG patterns also contribute to the development of force. These factors lead to the conclusion that the inferences of muscle force based on EMG should be made with caution [17]. Mathematical models and interpretation guides to infer force measurements in extremity skeletal muscle are available and provide a reasonable linear EMG–force relationship under isometric conditions [18]. Interestingly, anterior deltoid activity was correlated with experience level, laryngoscopy method, and table inclination, indicating that the efficient use of muscles requires an adequate combination of these three variables.

Although we recognize that multiple factors affect the force profile of the upper extremity during laryngoscopy, our study successfully isolated the effect of the anterior deltoid muscle as the main muscular group involved in laryngoscopy when conditions related to table inclination change. Caldiroli et al. found reduced muscle activity of posterior deltoid, brachioradialis, upper trapezius, and anterior deltoid with the use of Glidescope® in comparison with DL with Macintosh blade [19]. We show that anterior deltoid, biceps brachii, and posterior deltoid muscle activation is higher with direct laryngoscopy compared to videolaryngoscopy. Muscle activation of the same muscle groups is higher with C-MAC® compared to Glidescope®. Our study subjects, on average, generated high EMG activity using DL at 0° and moderate activity at 15° and 30° compared to the other two devices. These findings agree with Caldiroli’s results. Our study also showed that flexor carpi radialis muscle activation does not change with different laryngoscopic methods or different table inclination angles. We believe that this finding is related to the application of a force vector to the laryngoscope handle when the proper technique is employed. The force vector, in this case, has an upward and anterior direction, whereas a wrist rotation (by flexor carpi radialis activation) would characterize an inadequate technique [20].

Operator’s experience performing laryngoscopic procedures is a factor that affects the use of different muscle groups and the quality of the glottic view. Experience entails a better application of force during the procedure and an adequate posture with optimization of ergonomic variables [21]. On the other hand, Smith et al*.*showed that a favorable ergonomic position during laryngoscopy is associated with decreased muscle activation, fatigue, and self-reported pain [11]. Caldiroli’s study has limited generalizability of results as only ten anesthesiologists, all experienced using the Macintosh blade and Glidescope®, participated. It is unknown if similar findings would apply to anesthesiologists with varying degrees of experience and training [4]. We included participants with different levels of experience in our study. We did not find differences in muscle activation between years of experience; however, the time to intubation was longer for novices (PGY-1 and CA-1 residents).

Experience is necessary to turn a resident into a proficient laryngoscopist. Mulcaster et al*.* found that 47 ± 11.2 procedures are required to successfully intubate a patient in less than thirty seconds, whereas Konrad et al*.*described a learning curve with 90% success after 57 intubations [22, 23]. Compared with other studies, our results are within the time range of previous studies (34–206 s) [24]. We evidenced that only after a resident finishes their CA-1 year, the intubation times are comparable to those exhibited by more experienced groups. This contrasts with Mulcaster’s and Konrad’s studies, as CA-1 anesthesiology residents perform many more than sixty intubations during a year.

DL is associated with a lower success rate and longer intubation time compared with Glidescope® [24, 25]. Adequacy of vocal cord visualization has been traditionally evaluated with the Cormack-Lehane classification; however, we used POGO score to distinguish situations with small and large degrees of partial glottis visibility [26]. Our study found that the subjective POGO scores seen with DL were lower compared with objective POGO videolaryngoscopy scores. Although it is difficult to compare objectively measured POGO with subjective measures provided by the laryngoscopist, the difference in POGO objectively measured favors Glidescope® over C-MAC® to achieve the best view of the vocal cords.

Reduced peak and average forces applied to the airway during laryngoscopy with Glidescope® in comparison with Macintosh blade have been reported. However, as the duration of the procedure increases, the impulse force is comparable between the two devices [27]. Practitioners may face challenging intubations in settings where the conditions for adequate positioning of the patient are precarious. In these situations, the duration of laryngoscopy is expected to be prolonged, with the accompanying application of higher impulse force (product of force and time) [28]. In these less-than-ideal situations, elevating the head of the bed would be an easy maneuver to perform. We demonstrated that 15° and 30° of table inclination effectively reduce intubation time and the number of intubation attempts. Table inclination to 15° should be recommended as it provides the optimal effectiveness ratio to operator’s comfort.

On the other hand, less need to use optimization maneuvers means less risk of cervical extension or head movement during laryngoscopy. For this reason, the 15° table inclination would be ideal in trauma settings where cervical spine injury is concerning. Finally, the benefit of the 15° position is more pronounced when DL is utilized. Positioning the patient at 15^o^ should be a standard recommendation when videolaryngoscopes are not readily available. Tsan et al. evidenced that elevation of the head of the bed with DL is not inferior to videolaryngoscopy with Glidescope® and is devoid of adverse effects. All in all, table inclination seems to be a reasonable technique to facilitate laryngoscopy [29].

Our study has limitations. The rigidity and limited compliance of synthetic materials of a manikin differ from biological tissues encountered in a patient. Furthermore, manikin performance has been shown to be unequal amongst different manufacturers, thereby limiting the generalizability of our results [30]. Despite the heterogeneity of the participants in our study, only one manikin was used to provide consistency to the collected data since our aim was not to optimize learning but rather to evaluate current skills. Our study design limited laryngoscopy to a situation with an expected easy intubation. We would expect that the benefit obtained with changes in table position and use of different laryngoscopes to be experienced in difficult situations as well; however, this is difficult to affirm based solely on our results. Learning bias may have affected our results, as laryngoscopy was repeated at different inclinations and with different devices. However, the success rate was already high on the first attempt and carried on the following attempts, which would make this concern less problematic.

Furthermore, laryngoscopic view is just one component of securing the airway and a good laryngoscopic view does not necessarily mean easy intubation. Finally, under controlled conditions, to isolate the effect of table inclination, POGO should remain constant between operators. In our study, the operator was given the liberty to achieve the best possible glottic view, using the necessary upper extremity muscle force (and electrical activation). Although this variable POGO/muscle activation may make interpretation of results more difficult, this situation approximates what laryngoscopists encounter in a clinical setting.

## Conclusions

In conclusion, table inclination to a 15° angle reduces intubation time and need of optimization maneuvers and is associated with higher operator’s comfort levels compared to 0° and 30° inclination in a simulated scenario using a manikin. Anterior deltoid muscle activation is affected by both laryncoscopy method and table inclination, with the worst combination being DL at zero-degree of table inclination. Experience of the operator also affects glottis view and muscle activation. Vocal cord visualization is better with videolaryngoscopy compared to DL. Different upper extremity muscle groups are activated during laryngoscopy, with the anterior deltoid muscle exhibiting significantly higher activation levels with DL at zero-degree table inclination. Further research is needed to confirm the validity of our results in different settings and to evaluate the effect of different table inclination angles on intubation success rate in clinical situations.

## Data Availability

The datasets generated and analyzed during the current study are not publicly available due to subjects privacy concerns but are available from the corresponding author on reasonable request.

## Abbreviations

DL: Direct laryngoscopy
EMG: Electromyography
MVIC: Maximum voluntary isometric contractions
POGO: Percentage of glottis opening
POGO-subj: Subjective percentage of glottis opening
POGO-obj: Objective percentage of glottis opening
RMS: Root-mean-squared
